# Comparative precision of laboratory methods

**DOI:** 10.1155/S1463924687000221

**Published:** 1987

**Authors:** K. F. Yee

**Affiliations:** Syntex Research Centre Heriot-Watt University Edinburgh EH14 4AS UK


Journal of Automatic Chemistry/*Journal of Clinical Laboratory Automation*, Vol. 9, No. 2 (April–June 1987), pp. 100–101

### Comparative precision of laboratory methods

**K. F. Yee**
*Syntex Research Centre, Heriot-Watt University, Edinburgh EH14 4AS, UK*

### Introduction

Different quantitation techniques or methods of measurement are sometimes employed to measure the same substance in a laboratory. Higher precision, i.e. lower variability, is one of the criteria for selecting a better measurement method. This could be the case when one wants to compare a new (test) method to a standard (reference) method. When independent samples are used between methods, then it is well known that one can use the F-test [1] to compare their variances. However, when the same samples are employed for both quantitative methods, the F-test is inappropriate for comparing the between sample variation and could fail to detect the more precise method.

The appropriate statistical test was proposed by Pitman [2], [1] in 1939. This note illustrates the use of Pitman's test for comparative precision in the situation where the same samples are used for two laboratory quantitative methods.

### Method

Denote by $SD_i$ the standard deviation of the quantitation method $i$ ($i = 1,,2$) from $N$ samples, and the variance ratio between the two methods:

$$F = (SD_1/SD_2)^2 \qquad (1)$$

If the $N$ samples for method 1 are separate (independent) samples from those for method 2, then the F value in equation (1) behaves like an F-distribution with $(N-1)$ and $(N-1)$ degrees of freedom. One can therefore use this statistic for comparing their precision. However, if the same $N$ samples are used for both methods, $SD_1$ and $SD_2$ are correlated and the F value is no longer distributed as an F-distribution. A modified test by Pitman is as follows:

$$t = (F-1)/[(N-2)/4F(1-r^2)]^{1/2} \qquad (2)$$

is a $t$-distribution with ($N$-2) degrees of freedom, where $r$ is the correlation coefficient between the two methods and F is defined in equation (1).

### Example

To illustrate the Pitman's test an example has been taken from Griffiths *et al.* [3]: the sodium levels of 21 patient serum specimens were analysed by Beckman Astra-8 and flame photometry methods. The raw data are reproduced in table 1. From equation (1) the variance ratio:

$$F = (6{\cdot}725/5{\cdot}963)^2 = 1{\cdot}272$$

*Table 1. Raw data for Na by two quantitative methods (see Griffiths et al., 1986 [3]).*

| Sample | Astra | Flame |
|---|---|---|
| 1 | 129 | 130 |
| 2 | 140 | 139 |
| 3 | 135 | 137 |
| 4 | 139 | 138 |
| 5 | 132 | 131 |
| 6 | 140 | 139 |
| 7 | 138 | 137 |
| 8 | 136 | 137 |
| 9 | 135 | 135 |
| 10 | 144 | 145 |
| 11 | 142 | 142 |
| 12 | 140 | 139 |
| 13 | 119 | 121 |
| 14 | 134 | 135 |
| 15 | 151 | 149 |
| 16 | 139 | 138 |
| 17 | 134 | 133 |
| 18 | 142 | 141 |
| 19 | 146 | 143 |
| 20 | 145 | 143 |
| 21 | 141 | 142 |
| Mean | 138·1 | 137·8 |
| SD | 6·725 | 5·963 |
| Correlation coefficient ($r$) | | 0·9844 |
| No. | | 21 |

Had we used the F-test here, this statistic would not be statistically significant (probability >0.50), i.e. the two methods were equally precise. (The critical value of F at 0.05 level with 20 and 20 degrees of freedom = 2.46.) However, from equation (2):

$$t = (1{\cdot}272-1)/[(21-2)/4 \times 1{\cdot}272\ (1-0.9844^2)]^{1/2}$$
$$= 2{\cdot}987 \qquad \text{with 19 degrees of freedom}$$

is highly significant (probability <0.008), i.e. the flame photometry was more precise than the Astra method. (The critical value of $t$ at 0.05 level with 19 degrees of freedom = 2.093.) Here one can see that by ignoring the information that the data come from the same samples, one can fail to detect a superior method with respect to the precision by employing the F-test.

### Discussion

The problem of comparing variabilities discussed so far has been concerned with the situation where only a single measurement is available per sample per method. Where there are equal replications for each sample, both the between sample and within sample variations should be examined.

For the between sample variation (the same variability discussed in this note so far), one can take the average values over replicates and apply the Pitman's test on the sample averages. The individual replications do not enter into the statistical test directly.

The within sample variation measures the repeatability of the quantitation method on the same sample. Unlike the between sample variations, the within sample variations are not correlated between methods. In this instance one can use the F-test again for comparing the within sample variations. The statistical model with replication within sample is given in the Appendix.

### Appendix

The model:

$$y_{ijk} = \mu_{jk} + \eta_i + \tau_{ij} + \varepsilon_{ijk}$$

where $y_{ijk}$ is the determination from sample $i$ ($i = 1, \ldots, N$), quantitation method $j$ ($j = 1,2$) and replicate $k$ ($k = 1, \ldots, R$). $\mu_{jk}$ is the mean response of method $j$ and replicate $k$, and $\eta_i$, $\tau_{ij}$ and $\varepsilon_{ijk}$ are the residual error terms due to sample $i$, method $j$ and replicate $k$ respectively. Further assumptions are made on the residual error terms such that they are mutually independent and normally distributed:

$$\eta_i \sim N(O, \sigma_S^2)$$
$$\tau_{ij} \sim N(O, \sigma_{Mj}^2) \quad \text{and}$$
$$\varepsilon_{ijk} \sim N(O, \sigma_{Wj}^2)$$

Denote by $\bar{y}_{ij}$ the mean of sample $i$ and method $j$ over the $R$ replicates (i.e. $\bar{y}_{ij} = \sum_k y_{ijk}/R$) etc., then the between sample variance for method $j$:

$$\text{var}\ (\bar{y}_{ij}) = \sigma_S^2 + \sigma_{Mj}^2 + \sigma_{Wj}^2/R$$

and can be compared by the Pitman's test (using $\bar{y}_{ij}$).

$\sigma_{Wj}^2$ is the within sample variance, i.e. the variation between determinations due to replication. It is estimated by:

$$S_{Wj}^2 = \sum_{i,k} (y_{ijk} - \bar{y}_{ij} - \bar{y}_{jk} + \bar{y}_j)^2/(N-1)\ (R-1)$$

with $(N-1)\ (R-1)$ degrees of freedom. The F-test can be used here to compare $S_{Wj}^2$'s:

$$F = S_{W1}^2/S_{W2}^2$$

is distributed as an F-distribution with $(N-1)\ (R-1)$ and $(N-1)\ (R-1)$ degrees of freedom.

### References

1. Snedecor, G. W. and Cochran, W. G. *Statistical Methods* (The Iowa State Uni. Press, Iowa, 1976), p. 116, and p. 195.
2. Pitman, E. J. G., *Biometrika*, **31** (1939), 9.
3. Griffiths, W. C., Camara, P., Diamond, I. and Pezzullo, J. C., *Journal of Automatic Chemistry*, **8** (1986), 147.

**EUROSENSORS — 3RD CONFERENCE ON SENSORS AND THEIR APPLICATIONS**

*Eurosensors will take place at Cavendish Laboratory, Cambridge from 22 to 24 September 1987*

The conference will provide a forum for the presentation and discussion of recent advances in the sensor field. Topics to be covered include sensor designs, sensor packaging, materials for sensors and multisensor systems and software. The conference theme embraces physical, chemical and biological sensors and their applications.

*Invited papers*

Invited papers will include the following:

- Eurosensor scene (S. Middlehoek, Delft University of technology, The Netherlands).
- Sensor materials (W. E. Duckworth, Fulmer Research Institute, Slough, UK).
- Solid state chemical sensors (W. Gopel, Tubingen University, The Netherlands).
- Digital compensation of sensors (J. E. Brignell, University of Southampton, UK).
- Biosensors (C. R. Lowe, University of Cambridge, UK).
- Physiological sensors (D. Parker, University College Hospital, London, UK).
- Sensors in industrial metrology (B. E. Jones, Brunel University, UK).

*Euroworkshops*

- Sensors in the syllabus
- Software for sensor systems
- European community support for sensor projects

*Further information from Dr K. T. V. Gatton, Department of Physics, City University, Northampton Square, London EC1 0HB.*

